# Sulfated hyaluronan alters fibronectin matrix assembly and promotes osteogenic differentiation of human bone marrow stromal cells

**DOI:** 10.1038/srep36418

**Published:** 2016-11-03

**Authors:** Sarah Vogel, Simon Arnoldini, Stephanie Möller, Matthias Schnabelrauch, Ute Hempel

**Affiliations:** 1 Institute of Physiological Chemistry, Carl Gustav Carus Faculty of Medicine, Technische Universität Dresden, Fiedlerstrasse 42, 01307 Dresden, Germany; 2 Laboratory of Applied Mechanobiology, Department of Health Science and Technology, ETH Zurich, Vladimir-Prelog-Weg 4, 8093 Zurich, Switzerland; 3 Biomaterials Department, INNOVENT e. V., Pruessingstrasse 27B, 07745 Jena, Germany

## Abstract

Extracellular matrix (ECM) composition and structural integrity is one of many factors that influence cellular differentiation. Fibronectin (FN) which is in many tissues the most abundant ECM protein forms a unique fibrillary network. FN homes several binding sites for sulfated glycosaminoglycans (sGAG), such as heparin (Hep), which was previously shown to influence FN conformation and protein binding. Synthetically sulfated hyaluronan derivatives (sHA) can serve as model molecules with a well characterized sulfation pattern to study sGAG-FN interaction. Here is shown that the low-sulfated sHA (sHA1) interacts with FN and influences fibril assembly. The interaction of FN fibrils with sHA1 and Hep, but not with non-sulfated HA was visualized by immunofluorescent co-staining. FRET analysis of FN confirmed the presence of more extended fibrils in human bone marrow stromal cells (hBMSC)-derived ECM in response to sHA1 and Hep. Although both sHA1 and Hep affected FN conformation, exclusively sHA1 increased FN protein level and led to thinner fibrils. Further, only sHA1 had a pro-osteogenic effect and enhanced the activity of tissue non-specific alkaline phosphatase. We hypothesize that the sHA1-triggered change in FN assembly influences the entire ECM network and could be the underlying mechanism for the pro-osteogenic effect of sHA1 on hBMSC.

Glycosaminoglycans (GAG) are linear complex extracellular polysaccharides consisting of alternating disaccharide repeating units. They can exist solely or form proteoglycans by binding to a core protein. Heparin (Hep) is a natural highly sulfated polysaccharide, commonly isolated from mast cell or mucosa and used as an anticoagulant in the clinic^1^,^2^,^3^. The polymeric chain of Hep is constituted of a variously sulfated repeating sequence of uronic acid (L-iduronic acid, rarely D-glucuronic acid) linked through a 1,4-glycosidic bond to N-acetyl-D-glucosamine. Natural Hep is quite various and thus contains a large number of chains of different molecular weight. Heparan sulfate shares several chemical as well as structural features with Hep: It is also irregular, mostly less sulfated than Hep with a clustered sulfation pattern^2^,^4^. Both negatively charged sulfated GAG (sGAG) are able to interact with numerous proteins including growth factors and molecules of the extracellular matrix (ECM)^3^,^5^ thereby playing an important role for tissue engineering approaches^1^.

Several *in vitro* studies evaluated the importance of GAG and proteoglycans for osteoblast differentiation^6^,^7^,^8^,^9^,^10^. Synthetically sulfated GAG derivatives (sGAG) derived from non-sulfated hyaluronan (HA) as used in this study were previously shown to promote the osteogenic differentiation of human bone marrow stromal cells (hBMSC)^11^,^12^. These artificial sGAG derivatives altered several cellular processes such as various cell signalling pathways (e.g. BMP-2 (bone morphogenetic protein-2) and TGF-β1 (transforming growth factor-β1) signalling), proteins involved in endocytosis, cell-ECM-interaction, and ECM remodelling as well as matrix vesicle formation and composition^13^,^14^,^15^. Sulfation of GAG was found to be the underlying factor responsible for these effects as non-sulfated HA did not alter osteogenesis *in vitro*. In this study the synthetically low-sulfated HA derivative sHA1 (number of sulfate residues per disaccharide unit (degree of sulfation D.S.): 1.0–1.5) was chosen for further characterization of parental cellular mechanisms.

One major interaction partner of sGAG within the ECM is the glycoprotein fibronectin (FN). It represents one of the most abundant components of the ECM ubiquitously present both in mature and embryonic tissues as well as in the bone marrow niche^16^,^17^. FN is a disulfide-bonded homodimer (each 220 kDa) and is composed of three different types of domains (FNI, FNII and FNIII domains) (Fig. 1)^18^. Cells bind FN via RGD-binding integrins α_5_β_1_, α_V_β_1_ and α_V_β_3_^18^,^19^,^20^ and can apply mechanical forces generated by their contractile machinery and transferred via transmembrane integrins to FN^18^,^20^. Cellular forces can induce conformational changes in FN leading to an exposure of self-association sites for fibril assembly^20^,^21^,^22^,^23^. The structural assembly into stable FN fibrils is then based on intermolecular FN-FN interactions^24^,^25^,^26^.

FN is a binding partner of GAG, collagen I and other components of the ECM and facilitates network formation^26^,^27^,^28^. It homes three Hep binding regions located at FNI_2-4_, FNIII_4-6_ and FNIII_12-14_ (Fig. 1)^29^,^30^. Hep binding to FN in solution converts the FN dimer from a globular to an elongated structure and thus can enhance growth factor binding by presentation of cryptic binding sites^31^. Several studies revealed that FN is a key player in the commitment of BMSC to osteoblast lineage supporting BMSC adhesion and ultimately differentiation into osteoblasts^32^,^33^,^34^.

In the present study, we addressed the question whether the synthetically low-sulfated HA derivative sHA1 affects cellular FN matrix assembly and how this could be related to the pro-osteogenic effect of sHA1 on hBMSC^12^. Low-molecular weight HA and Hep served as controls for the experiments. Fluorescent-labeled sHA1 derivative was used to visualize the localization of sHA1 within the ECM. The influence of sHA1 on FN conformation within the hBMSC-deposited ECM was studied with a fluorescence resonance energy transfer (FRET) probe as a measure for molecular extension of FN^22^,^35^. Effects on FN formation were analyzed on mRNA level (real time PCR) and protein level (Western blot). A FN solubility assay according to Wierzbicka-Patynowski *et al.*^36^ characterized FN fibril matrix assembly status. Finally the effect of sHA1, HA and Hep on osteogenic differentiation of hBMSC was evaluated determining the activity of tissue non-specific alkaline phosphatase (TNAP).

## Results

### sHA1 and Hep colocalize with FN fibrils and induce a more extended conformation of fibrillary FN

To investigate localization behavior of GAG derivatives, hBMSC were cultured on tissue culture polystyrene plates (TCPS) in DMEM and were treated from day 1 until day 8 with ATTO565-labeled sHA1 and HA derivatives. When analyzing the GAG localization at day 8, the permanent treatment of hBMSC (d1-d8) with non-sulfated ATTO565-HA did not result in any specific structures (Fig. 2B). In contrast to that a fibrillary arrangement of ATTO565-sHA1 was seen, when hBMSC were permanently treated with the low-sulfated HA derivative (Fig. 2C). This situation however changed when ATTO565-sHA1 was removed at day 4 by exchange of medium (treatment d1-d4, Fig. 2D) confirming that a permanent presence of ATTO565-sHA1 (d1-d8) is required for a sustained fibrillary arrangement and that this arrangement is reversible.

Apart from the fibrillary sHA1 arrangement, a considerable amount of ATTO565-sHA1 got accumulated intracellularly and was detected in perinuclear vesicles (see Fig. 2C,D,G) indicating a putative endocytotic uptake mechanism^37^.

To see whether the fibrillary sHA1 arrangement occurred extracellularly, the hBMSC were removed from the ECM by decellularization with NH_4_OH, a decellularization protocol known to keep the endogenous ECM intact^38^. Figure 2E demonstrates that the fibrillary arrangement of sHA1 formed by hBMSC was stable even after decellularization which confirmed a specific extracellular action of sHA1.

Structural ECM proteins were immunofluorescently stained at day 8 after permanent treatment of hBMSC with ATTO565-GAG derivatives to identify putative interaction partners forcing sHA1 into such a fibrillary arrangement. FN (green fluorescence) showed a dense fibrillary network which strongly colocalized with ATTO565-sHA1 (red fluorescence) in yellow appearing structures (Fig. 2C,E) but did not show the same localization as the non-sulfated ATTO565-HA derivative (Fig. 2B). Since the ATTO565-sHA1 was permanently present in this approach it remains open whether sHA1 interacts with already assembled FN fibrils or whether it was incorporated into the structures during FN fibrillogenesis. To address this question, cells were treated with ATTO565-sHA1 for 24 h from day 7 until day 8. In this setting a colocalization of FN-fibrils with ATTO565-sHA1 was found (Fig. 2F) suggesting indeed a direct binding of sHA1 to preformed FN fibrils. Addition of both, fluorescently labeled FN (AlexaFluor488-FN) and ATTO565-sHA1 to hBMSC for 48 h revealed that ATTO565-sHA1 can interact also with newly formed, nascent FN fibrils (Fig. 2G).

Beside the synthetic sHA1 derivative, Hep was used as naturally sulfated control to analyze its FN binding *in vitro*. For this purpose, hBMSC were treated with 200 μg FITC-Hep/mL from day 1 till day 8 and samples were fixed and co-stained for FN at day 8 (Fig. 2H). The FITC-signal in the green channel (labeled in red) colocalized with FN fibrils in the red channel (labeled in green) within yellow appearing structures.

As depicted in Fig. 2C,E–G ATTO565-sHA1 and FN fibrils displayed a similar pattern suggesting colocalization of both components within the hBMSC-derived ECM. To assess this more into detail, a quantitative analysis of ATTO565-sHA1 (red channel) and AlexaFluor488-FN (green channel) colocalization (according to Fig. 2G) was performed using the colocalization plugin JACoP in Fiji Software^39^ (Fig. 3). As the signal intensities of green and red channel differ from each other, intensity independent colocalization values (Pearson and Manders’) were computed. For colocalization of ATTO565-sHA1 and AlexaFluor488-FN, a Pearson’s coefficient of 0.740 was calculated, which confirmed partial colocalization; a value of 1 would indicate 100% colocalization. Manders’ coefficients M1 and M2 showed similar values around 85% for each channel.

The localization studies thus showed that the herein used chemical coupling of ATTO-TEC fluorescent dyes to GAG derivatives was a suitable method to generate fluorescent labeled GAG derivatives for *in vitro* localization studies. As control to exclude possible artifacts from free dye molecules a double-labeled ATTO565-ATTO655-sHA1 derivative was used to confirm the stability of labeled sHA1-molecule. Therefore to assess labeling stability a quantitative colocalization analysis of the two dyes (as described above) confirmed nearby complete colocalization of ATTO565 and ATTO655 (see supplementary Fig. S1). Almost 100% colocalization of both dyes *vice versa* showed an adequately stable coupling of ATTO-TEC-molecules to sHA1 and hence confirmed the stability of the GAG chains as fluorescent labels were not separated by degradation. Additionally, free dye molecules of ATTO565-NH_2_ did not specifically interact with any cellular targets (data not shown) confirming that the sHA1-dye complex and not the free ATTO565-dye colocalized with FN fibrils.

Immunofluorescence staining indicated that ATTO565-sHA1 and FITC-Hep colocalize with FN fibrils that were assembled by hBMSC in presence of 200 μg sGAG/mL (Fig. 2C,H). The interaction of FN with Hep is known as FN has several Hep binding regions^40^,^41^,^42^ and its binding was shown to regulate FN conformation in matrix fibrils^31^. Binding and colocalization of the synthetically sulfated sHA1 derivative with FN fibrils assembled by hBMSC, however, was shown herein for the first time. To further investigate similarities between those two sGAG we assessed whether the sHA1 derivative exhibits comparable effects on FN conformation as Hep. FN conformation was probed via addition of small amounts of a double-labeled FRET-FN (FN donor acceptor: FN-DA) into the cell medium^21^,^22^. Images in Fig. 4A show representative color-coded FRET ratio (acceptor/donor) images of hBMSC-derived ECM after 72 h of incubation with FN-DA. The color code indicated the range of conformational states of FN fibrils from red (compact, FRET ratio of 1) to blue (highly stretched, FRET ratio of 0). The synthetically sulfated sHA1 derivative induced a decrease in FN FRET ratios similar to Hep, indicating a more extended FN conformation compared to the untreated control (CTRL). Our data of more extended FN fibers upon Hep treatment go in line with previously published results^35^. Other than sHA1 and Hep the non-sulfated HA did not affect FRET ratios and behaved comparably to the untreated control sample (CTRL). The box and whiskers plot in Fig. 4B summarizes mean FRET ratios calculated from four donors each in duplicate and confirms a significant shift towards lower FRET ratios by sHA1 and Hep in comparison to CTRL. No significant difference was seen between CTRL and HA treated samples. These changes were also reflected in histograms of average FRET ratios obtained from 10 independent images of one representative donor each in comparison to CTRL (grey lines; Fig. 4C). In all cases FRET values showed a similar spread and width of corresponding histograms demonstrating that a similar range of FN conformations is coexistent in FN fibrils within the ECM. However, the histograms of samples treated with Hep (green line) and sHA1 (red line) were clearly shifted towards lower FRET ratios compared to HA (orange line) and CTRL.

### sHA1, but not Hep, increases FN protein level and alters FN assembly status of hBMSC

Immunofluorescence images indicated that the presence of sHA1 might upregulate FN matrix assembly as the FN network seems denser and composed of more but thinner FN fibrils in comparison to the untreated control (see Fig. 2A,C). Neither HA nor Hep altered FN network in comparison to CTRL (see Fig. 2B,H).

To investigate the first qualitative observation more into detail, differences in FN matrix assembly were evaluated using the previously reported deoxycholate (DOC) solubility assay^36^. This assay discriminates between less assembled and thus DOC-soluble FN fibrils, and the DOC-insoluble fraction containing stably assembled FN fibrils only being dissolvable in sodium dodecyl sulfate (SDS) containing buffer. Samples for the DOC- and SDS-lysates of hBMSC were permanently treated for 8 days with Hep, sHA1 or HA, respectively, and the FN content of both lysates was analyzed via Western blotting (main bands at ~220 kDa) and densiometric evaluation of the signals (Fig. 5A–C¸ full length blots in supplementary Fig. S2). Quantitative Western blot data indeed confirmed our qualitative observation from immunofluorescence images as sHA1 treatment increased the total FN protein level about 2- to 3-fold in comparison to CTRL, HA and Hep. As seen in Fig. 5B, the increase of total amount of FN (sum of DOC- and SDS-fraction) is mainly the result of increased DOC-soluble, less assembled FN. This goes along with a sHA1-induced significant shift towards DOC-soluble FN (about 80% of total FN was DOC-soluble) in comparison to CTRL (about 40% of total FN was DOC-soluble) (Fig. 5C). In contrast to sHA1, Hep did neither affect FN protein level nor FN assembly status if compared to untreated control. Most likely, sulfation of HA was responsible for these effects as non-sulfated HA did not alter the total FN protein content and exhibited a distribution of FN fractions comparable to the untreated control. In summary, the characterization of FN assembly status showed that exclusively sHA1 induced a clear shift from strongly assembled DOC-insoluble to less assembled DOC-soluble FN fibrils.

To assess whether the overall higher FN protein content in cell lysates treated with sHA1 might be a result of an increased *fn1* gene expression real time PCR analysis was carried out at day 1 and day 8 of GAG treatment. PCR analysis showed that *fn1* mRNA levels were significantly increased by sHA1 at day 1 and later time points, whereas Hep and non-sulfated HA treated cells exhibited comparable *fn1* expression to untreated control (Fig. 5D).

### sHA1, but not Hep, increases TNAP activity in hBMSC

As seen, exclusively sHA1 increased FN protein level and altered FN matrix assembly status of hBMSC. To evaluate the consequences of that for hBMSC differentiation, the activity of TNAP, a recognized marker for early osteogenic differentiation^43^, was determined. sHA1 induced a significant increase of TNAP activity in hBMSC by about 3.5-fold compared to untreated control, HA and Hep (Fig. 6A). Several studies showed that sHA derivatives as a component of artificial collagen-based matrices revealed pro-osteogenic effects on hBMSC^11^,^12^. Figure 6B demonstrates that the effect of sHA1 and HA on TNAP activity was not influenced by the fluorescence label. Just as the effect of sHA1 on FN structure lasted as long as sHA1 was present (see Fig. 2C,D,F), also the effect of sHA1 on TNAP activity required the permanent treatment with sHA1 (Fig. 6C). The addition of sHA1 to hBMSC cultures at later time points (day 4 or day 7) resulted in a loss of the pro-osteogenic effect. All effects of GAG derivatives on TNAP activity were found in basic medium (without osteogenic supplements) and osteogenic medium (DMEM supplemented with β-glycerophosphate, ascorbate, dexamethasone), as well (see supplementary Fig. S3).

## Discussion

**Figure 1:**
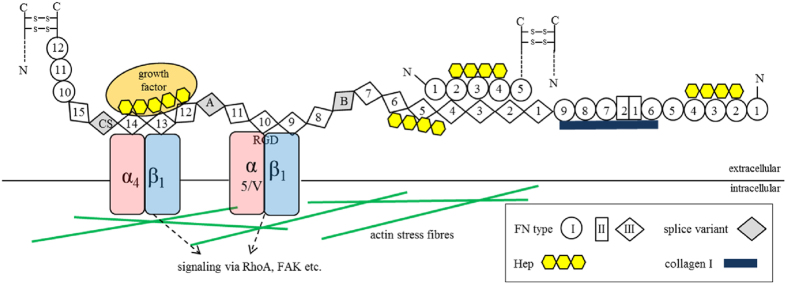
Domain structure and binding partners of FN. FN is secreted as a disulfide-bonded homodimer, which is composed of three types of repeating modules (FNI, FNII and FNIII) numbered serially. Splice variants may additionally contain extra domains A (EDA) or B (EDB) and a variable region CS (connecting segment) (all in grey). FN has several binding domains for interaction partners in the ECM like collagen I (dark blue) or growth factors (orange). Each FN chain has three Hep binding regions (yellow) located at FNI_2-4_, FNIII_4-6_ and FNIII_12-14_, respectively. The antiparallel, intermolecular interaction of FNI_1-5_ and FNIII_1-5_ is crucial for FN fibrillogenesis. The RGD binding site is located at FNIII_10_ and is recognized by integrins α_5_β_1_, α_V_β_1_ and α_V_β_3_ (red and blue). Several intracellular processes are directly affected by integrins through cytoskeletal adapter (actin in green) and signaling molecules as FAK (focal adhesion kinase) and small GTPases as RhoA.

**Figure 2:**
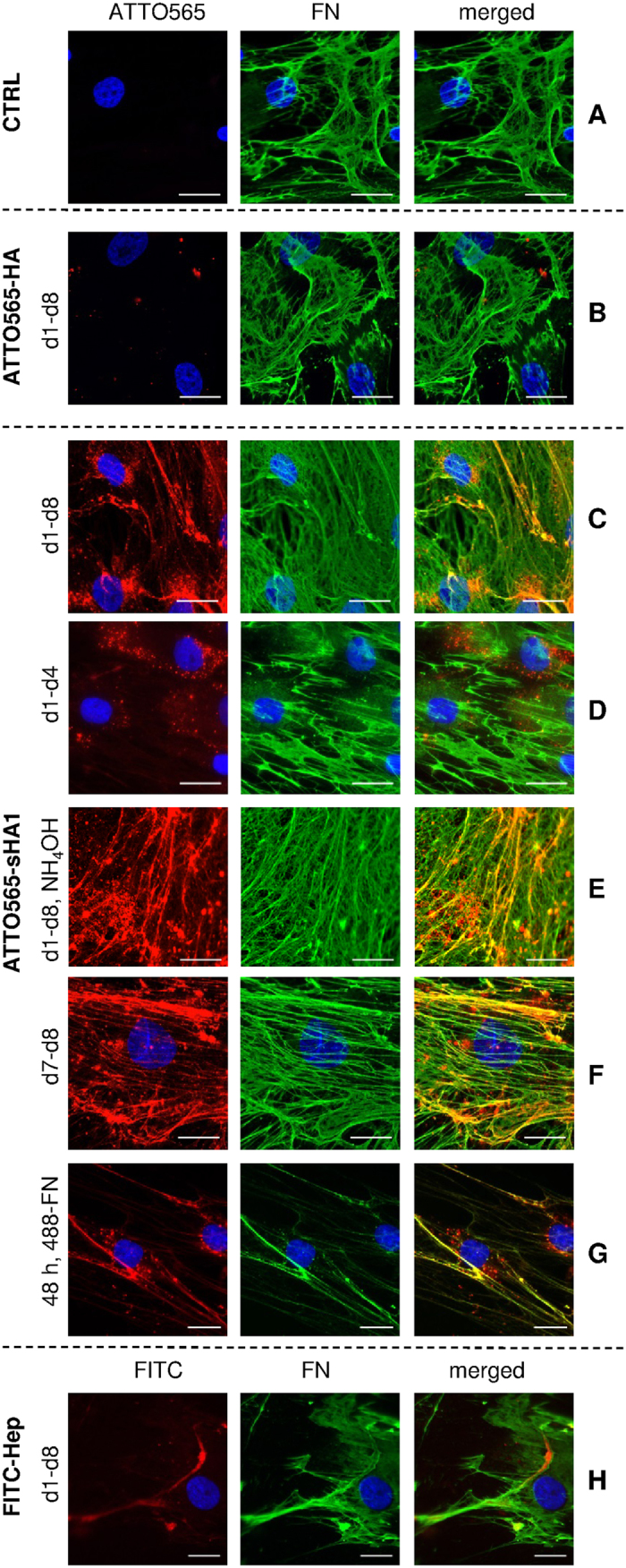
Localization of ATTO565-HA, ATTO565-sHA1 and FITC-Hep in hBMSC and impact on FN matrix assembly. hBMSC were cultured on TCPS (control, CTRL, **(A**). At day 8 after seeding the cells were washed, fixed and stained with mouse anti-human FN-IgG/AlexaFluor488-goat anti-mouse IgG (green fluorescence). The nuclei were visualized by DAPI staining (blue fluorescence). hBMSC were treated from day 1 until day 8 with 200 μg ATTO565-HA/mL (red fluorescence) (**B**) or 200 μg ATTO565-sHA1/mL (**C**) and stained at day 8 as described before. hBMSC were treated from day 1 until day 4 (**D**) with 200 μg ATTO565-sHA1/mL, fixed at day 8 and stained as described before. At day 8, samples, which were treated permanently with ATTO565-sHA1, were decellularized as described and the remaining ECM was fixed and stained for FN (**E**). hBMSC were treated from day 7 until day 8 (**F**) with 200 μg ATTO565-sHA1/mL and stained at day 8 as described before. hBMSC were treated with 200 μg ATTO565-sHA1/mL, 5 μg AlexaFluor488-FN/mL and 45 μg plasma-FN/mL. After 48 h cells were fixed and nuclei were stained with DAPI (**G**). hBMSC were treated with 200 μg FITC-Hep/mL (green fluorescence, labeled in red). At day 8 after seeding the cells were washed, fixed and stained with mouse anti-human FN IgG/AlexaFluor568-goat anti-mouse IgG (red fluorescence, labeled in green) and DAPI (blue) (**H**). Scale bars A-D and G-H 50 μm, E-F 20 μm.

**Figure 3:**
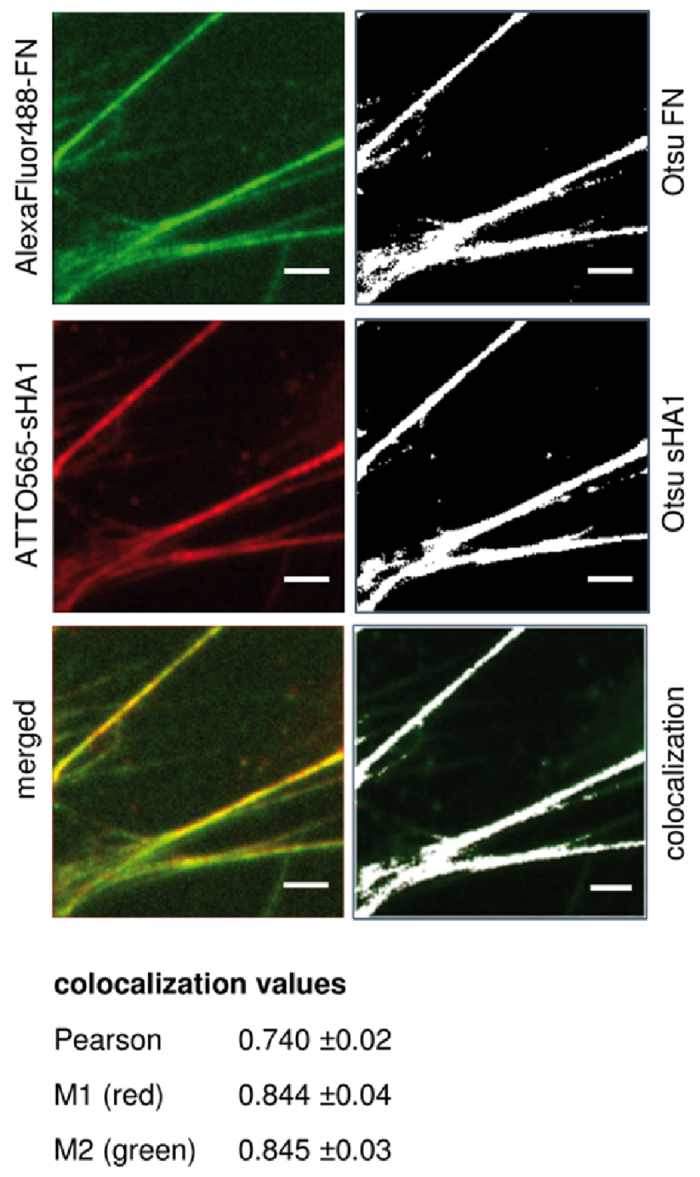
Colocalization analysis of FN and sHA1. hBMSC were cultured on TCPS. From 24 h after seeding, hBMSC were treated with 5 μg AlexaFluor488-FN/mL (green fluorescence), 45 μg plasma-FN/mL and 200 μg ATTO565-sHA1/mL (red fluorescence) simultaneously. After further 48 h cells were fixed, stained with DAPI and embedded in Mowiol 4-88 as described before. Yellow fluorescence indicated overlaid structures of AlexaFluor488-FN and ATTO565-sHA1 (left panel). To identify and quantify colocalization, threshold was set using Otsu’s method and images were visualized in false colors (right panel; signal in white, background in black) and subjected to JACoP Plugin in Fiji Software. Colocalized structures are colored in white (right panel). Scale bar 5 μm. The data of colocalization analysis of 15 randomly chosen images (values as mean ± SEM (standard error of the mean)) are summarized underneath.

In this study, we describe for the first time the impact of sulfated HA derivative on FN matrix assembly in hBMSC-derived ECM. Fluorescently labeled sulfated sHA1 derivative (ATTO565-sHA1) was found to be present in the ECM of hBMSC showing a fibrillary arrangement (Fig. 2C–G) similar to that seen with fluorescently labeled Hep (Fig. 2H). Co-staining experiments demonstrated a strong colocalization of ATTO565-sHA1 with FN within the extracellular network, which was confirmed by a quantitative colocalization analysis (Fig. 3). This seems reasonable, as FN has three Hep binding regions, which also might be electrostatically favorable for the interaction with sHA1 but not with non-sulfated HA. Our localization studies with ATTO565-sHA1 and FITC-Hep suggest an electrostatics-driven interaction of both sGAG with extracellular FN fibrils (Fig. 2C,H), whereas non-sulfated ATTO565-HA did not colocalize with FN (Fig. 2B). The sHA1-FN colocalization was detectable as long as the sHA1 was present in the ECM (Fig. 2C), and was reversible after removal of sHA1 (Fig. 2D). This goes in line with previously published findings reporting that ionic sGAG-FN interactions are reversible under physiological conditions and that surface diffusion and frequent un- and re-binding events enable one sGAG chain to interact with multiple FN molecules^44^. sGAG binding to these regions may compete with other molecules within the crowded environment of a FN fibril^19^ as all three Hep binding regions have multiple binding partners (see Fig. 1).

Qualitative observations from Fig. 2 suggested a strong effect of sHA1 but not of Hep on FN network structure. Presence of sHA1 for 8 days led to a FN matrix morphology with an increase of thinner fibrils when compared to untreated control. Despite a proven interaction of FN with Hep similar to sHA1, Hep had no visible effect on FN network structure. This difference is likely based on the different molecular structure of both sGAG: The synthetic sHA1 derivative is composed of consistent disaccharide units with a regular and equal sulfation pattern over the whole polymer length^45^ (see Fig. 7), whereas the natural Hep consists of variable disaccharide units with diverse uronic acid residues and has a more randomly distributed, clustered sulfation pattern^2^,^4^,^46^ (see Fig. 8).

**Figure 4:**
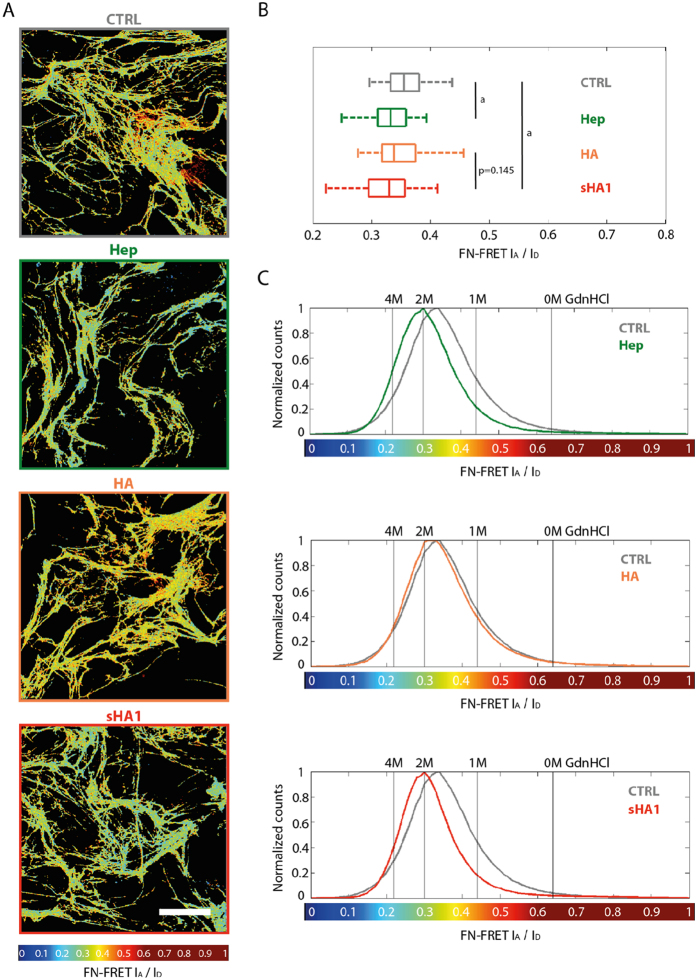
Impact of GAG derivatives on FN conformation. hBMSC were cultured on TCPS and incubated with 45 μg plasma-FN/mL and 5 μg FN-DA/mL from 2 h after seeding. After 24 h, the cells were treated with Hep, HA or sHA1 (200 μg/mL each). Untreated cells served as control (CTRL). After 48 h of GAG incubation, samples were fixed with 4% (w/v) paraformaldehyde in PBS for 10 min and embedded in Mowiol 4-88. For FRET analysis FN donor and acceptor channels were visualized (**A**). The FRET color scheme represents the relative conformational changes on FN fibrils within a color range of red (compact) to blue (highly stretched). Scale bar 50 μm. Box and whiskers plot of mean FRET ratios calculated from four donors each in duplicate is given in (**B**). Statistical significances between CTRL and Hep respectively CTRL and sHA1 are indicated with a (p < 0.05). Representative histograms of FRET ratios obtained from 10 randomly chosen images after treatment with Hep (green line), HA (orange line) and sHA1 (red line) in comparison to untreated control (gray line) are shown in (**C**) (n = 1). The vertical lines represent FRET ratios of FN-DA in solution which was denatured in 1, 2 or 4 M GdnHCl, respectively.

To quantify the effect of sHA1 on the FN matrix assembly status we used a solubility assay, which revealed, that sHA1 induced a significant shift from strongly assembled, DOC-insoluble to less assembled, DOC-soluble FN fibrils (Fig. 4). The conversion into DOC-insoluble FN fibrils is based on strong, non-covalent protein-protein interactions^47^ and other factors such as extracellular transglutaminase-catalyzed crosslinking activity^48^ and intermolecular disulfide bonding, catalyzed by the intrinsic protein disulfide isomerase activity of FN^49^. All these factors might be affected by sHA1, and thus prevent the formation of DOC-insoluble FN fibers.

HA sulfation directly affected ECM composition as presence of sHA1 increased the total FN protein level (Fig. 4), which was shown to be primarily based on an sHA1 induced elevated expression of *fn1*, revealed by PCR analysis. Non-sulfated HA did neither affect the *fn* gene expression nor its protein level suggesting that sulfation of HA is the driving force required for this effect. Also Hep had no impact on FN protein or *fn1* RNA level, suggesting a sHA1-specific mechanism, potentially based on the constant sulfation pattern in this molecule. Another explanation for the extracellular accumulation of FN fibrils could be a reduced enzymatic FN matrix degradation by proteolytic enzymes. Previous studies showed that synthetic sHA derivatives reduced the activity of matrix metalloproteinase-2 (MMP-2), one of the FN-cleaving proteolytic enzymes^14^,^15^. This MMP reduction might be triggered by the increased sHA-induced formation of MMP inhibitor TIMP-3 (tissue inhibitor of metalloproteinase-3) that could further contribute to FN accumulation via blocking MMP-related matrix degradation^15^.

FN conformation within ECM fibrils was assessed via a FRET based molecular tension probe for FN. Presence of sHA1 led to an extension of FN, similar to previously described effects of Hep on FN^35^, while non-sulfated HA did not affect FRET ratios in comparison to the untreated control (Fig. 3). Thus the actual FN conformation is a result of sGAG binding in combination with cell-induced mechanical strain^22^,^35^,^41^,^50^, binding of both sGAG (synthetic sHA1 as well as natural Hep) to FN was shown to induce a conformational extension within FN fibrils deposited in hBMSC-derived ECM. The non-sulfated HA did not show any visible effect on FN conformation which goes in line with previously published results^31^. This is of special relevance as FN has many cryptic binding sites which can be unmasked and exposed by sGAG binding^31^,^44^,^51^. These cryptic sites include various FN self-assembly sites whose exposure is needed to induce FN fibrillogenesis^52^. Such conformational changes in FN molecules can have drastic impacts as they strongly regulate FN function, e.g. growth factor binding^31^,^44^.

**Figure 5:**
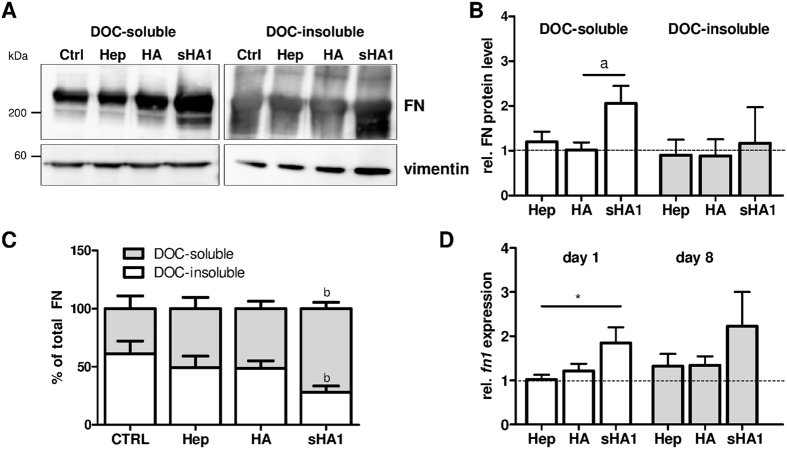
Influence of sHA1 on FN protein content and fn1 gene expression. hBMSC were cultured on TCPS and treated permanently from day 1 until day 8 with 200 μg Hep/mL, 200 μg HA/mL or 200 μg sHA1/mL. Untreated cells served as control (CTRL). At day 8, cells were lysed in DOC-buffer. The DOC-insoluble proteins were subsequently solubilized in SDS buffer. Both fractions were subjected to a 4–15% (v/v) SDS PAGE gradient gel and analyzed by Western blotting for FN protein content (main bands at ~220 kDa) using vimentin (bands at ~55 kDa) as internal control. FN and vimentin were detected using monoclonal mouse anti-human IgGs/HRP-conjugated anti-mouse IgG on the same blot and subsequent chemiluminescence detection. Image in (**A**) presents two representative cropped Western blots of DOC-soluble and DOC-insoluble fractions from the same experiment processed in parallel (full length blots in supplementary Figure S3). Main bands from FN at ~220 kDa were identified from smeary bands from DOC-insoluble fraction. The diagram in (**B**) shows the densiometric quantification of Western blot signals of both fractions using ImageQuant software. Values of densiometric evaluation of FN were related to values of vimentin and then normalized to untreated control (CTRL, indicated with dotted line). Values are shown as mean ± SEM and significant differences between sHA1 and HA are indicated with a (p < 0.05). n = 4. The calculation of percentage of total FN in DOC-insoluble and DOC-soluble fractions is given in (**C**). Values are shown as mean ± SEM and significant differences between sHA1 and CTRL are indicated with b (p < 0.01), n = 4. At day 1 and 8 cells were lysed for RNA preparation and analyzed after reverse transcription for expression of *fn1* by real time PCR (**D**). Comparative quantification was normalized to *rps26.* Values were normalized to CTRL for each time point (indicated with dotted line) and are shown as mean ± SEM. Significant differences between Hep and sHA1 at day 8 are indicated with a (p < 0.05). n = 3.

**Figure 6:**
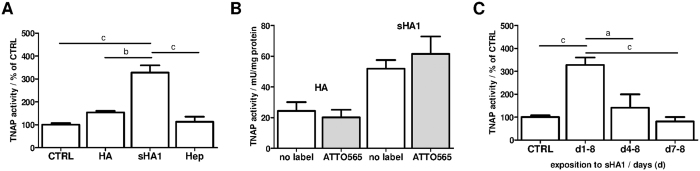
Impact of GAG derivatives on TNAP activity. hBMSC were cultured on TCPS (CTRL) and treated with dissolved GAG derivatives as described. TNAP activity was determined from cell lysates at day 8. Diagrams show calculated values as mean ± SEM. (**A**) hBMSC were treated with 200 μg GAG derivative/mL for 8 days. Diagram shows TNAP activities calculated from four different donors and untreated control (CTRL) was set to 100%. Significant differences between sHA1 and CTRL, HA or Hep are indicated with c (p < 0.001) and b (p < 0.01). (**B**) hBMSC were treated with 200 μg HA or sHA1/mL or with the equivalent ATTO565-labeled derivative for 8 days. n = 3. (**C**) hBMSC were exposed to 200 μg sHA1/mL for different time frames (d1-8, d4-8, d7-8) and TNAP activities were determined at day 8. Untreated control (CTRL) was set to 100%. Significant differences between (d1-8) and CTRL, (d4-8) or (d7-8) are indicated with c (p < 0.001) and a (p < 0.05). n = 3.

The sHA1-specific effect on FN matrix assembly status and protein level was in a last experiment linked to an osteogenic lineage commitment of hBMSC: Early osteogenic marker TNAP was increased only in presence of sHA1 and not HA or Hep (Fig. 5A). Furthermore a permanent presence of sHA1 from day 1 after seeding (d1-8) was required for the osteogenic commitment and thus on TNAP activity (Fig. 5C). sGAG, in particular Hep and heparan sulfate, have been reported to promote osteogenesis of BMSC^6^,^35^,^53^. Effects have been strongly dependent on cellular systems, detailed sGAG properties (doses, molecular weight, number of sulfate residues per disaccharide unit (degree of sulfation D.S.), sulfation pattern) and protocols (e.g. for ECM fixation).

This study demonstrated similarities of synthetic sHA1 and natural Hep concerning interaction with FN resulting in conformational stretching of FN in cell-derived ECM fibers. However, the two sGAG revealed different effects on FN protein level and matrix assembly: Here, it is shown for the first time that sHA1, but not Hep, induced increasing levels of *fn1* RNA, FN protein level and amount of DOC-soluble FN fibrils. We could link these findings with an increased TNAP activity indicating a strong pro-osteogenic effect of sHA1 on hBMSC. We hypothesize that the change in ECM network triggered by sHA1 is the underlying factor altering growth factor binding and thus outside-in signaling, hence offering a possible explanation for the pro-osteogenic effect of sHA1 on hBMSC.

## Materials and Methods

### Preparation of synthetic HA derivatives

Natural high molecular weight HA was obtained from Aqua Biochem (isolated from *Streptococcus*, M_W_ (LLS, Laser Light Scattering) = 1.1∙10^6^ g/mol, PD = 4.8).

A degraded, low-molecular weight HA, was prepared as previously described to serve as reference material for *in vitro* experiments since the molecular weight of natural HA precursor is decreased during sulfation^45^,^54^,^55^,^56^ HA derivatives with a degree of sulfation (D.S., number of sulfate residues per disaccharide unit) of 1.0–1.5 referred to as low-sulfated HA derivative sHA1 (Table 1).

**Figure 7:**
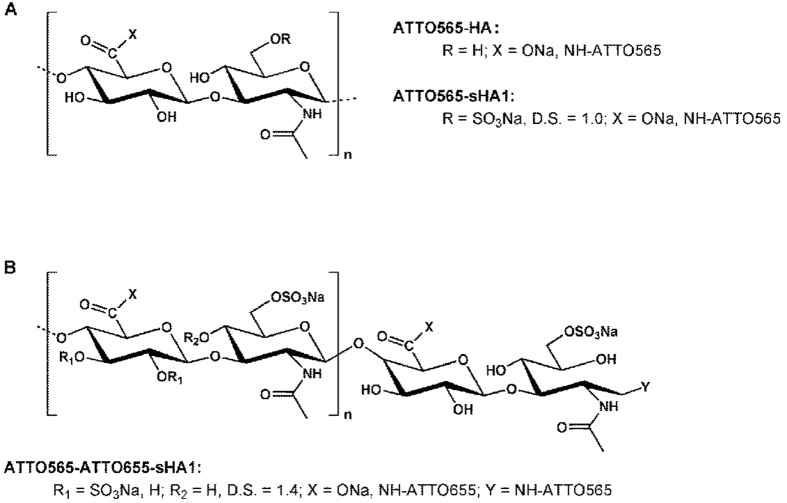
Chemical structure of synthetically sulfated and ATTO-labeled HA derivatives. HA consists of alternating disaccharide units (D-glucuronic acid and N-acetyl-glucosamine) connected by glycosidic bonds. The sHA1 derivative (D.S. 1.0–1.5) is preferentially sulfated at C6 of the N-acetyl glucosamine unit. The labeling of HA and sHA1 was performed with ATTO565-NH_2_ and resulted in a side-on modification of carboxylic groups (**A**). The double-labeled ATTO565-ATTO655-sHA1 derivative was generated by end-on modification with ATTO565-NH_2_ followed by side-on functionalization with ATTO655-NH_2_ (**B**).

The functionalization of HA-derivatives (HA, sHA1) with fluorescence dye ATTO565-NH_2_ was carried out at the carboxyl groups of the D-glucuronic acid unit of HA (side-on functionalization) using a conventional EDC/NHS coupling protocol (Fig. 7, Table 1)^56^,^57^. The double-labeled ATTO565-ATTO655-sHA1 derivative was prepared by coupling ATTO565-NH_2_ to the reducing end of sHA1 via an azomethine bond and subsequent reduction of the C=N double bond (end-on functionalization), followed by coupling of ATTO655-NH_2_ at the carboxyl group of the GAG using EDC/NHS (1-Ethyl-3-(3-dimethylaminopropyl)carbodiimide/N-hydroxy succinimide).

### Hep derivatives

**Figure 8:**
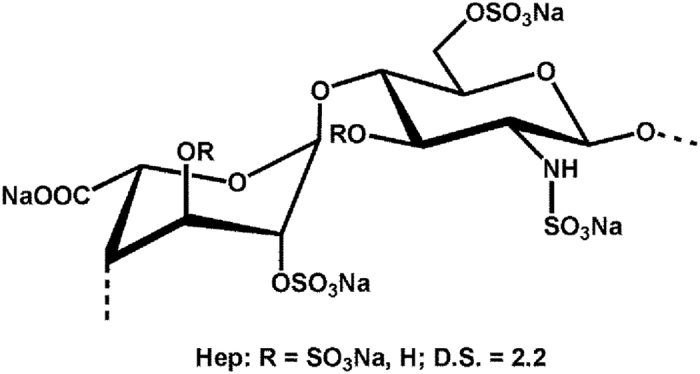
Disaccharide structure of Hep. The polymeric chain is composed of repeating disaccharide unit of D-N-acetyl glucosamine and uronic acid (L-iduronic acid, rarely D-glucuronic acid) linked by 1,4-glycosidic bond.

Sodium Hep derivatives (D.S. of 2.2; disaccharide structure in Fig. 8) derived from porcine intestinal mucosa. Hep (Sigma-Aldrich) had a molecular weight range from 6–30 kDa; fluorescent FITC (5-fluorescein isothiocyanate)-Hep (λ_ex_ = 491 nm, λ_em_ = 516 nm; Polysciences) had a molecular range from 17–19 kDa.

### Cultivation of hBMSC

hBMSC were isolated from bone marrow aspirates at the Bone Marrow Transplantation Center of University Hospital Dresden, according to Oswald *et al.*^58^. The study was approved by the local ethics commission of the University of Technology Dresden (ethics vote no. EK114042009) and the donors (females and males, average age 28.2 years) were informed about the procedures and gave their consent. Methods were carried out in accordance with the relevant guidelines (good scientific practice according to guidelines of German Research Foundation (DFG)).

For experiments, 7,000 hBMSC/cm^2^ were plated onto tissue culture polystyrene plates (TCPS). Dulbecco’s Modified Eagle’s medium low glucose (DMEM) with 10% (v/v) heat-inactivated fetal calf serum (FCS), 20 μg streptomycin/mL and 20 U penicillin/mL was used. hBMSC were treated with 200 μg GAG/mL. If not mentioned otherwise GAG were added 2 h after seeding and again with each change of medium performed twice a week.

For higher induction of TNAP (tissue non-specific alkaline phosphatase), osteogenic supplements were added at day 4 to the medium^11^,^12^,^13^.

### Immunofluorescence staining

Immunofluorescence staining was performed as previously described^13^,^14^,^55^. Fixed cells were incubated with primary monoclonal mouse-anti-human FN antibody (1.25 μg/mL; BD Biosciences) followed by incubation with secondary antibody AlexaFluor488- or AlexaFluor568-conjugated goat anti-mouse IgG and 0.2 μg DAPI/mL (4′,6-diamidino-2-phenylindole). Afterwards, the cells were embedded in Mowiol 4–88 and visualized using an Olympus IX70 fluorescence microscope.

For detection of extracellular localization of sHA1, cells were removed from ECM prior fixation step by incubation in 25 mM NH_4_OH for 20 min at room temperature keeping the endogenous ECM intact.

### Analysis of nascent FN fibrils

For demonstration of incorporation of sHA1 into nascent FN fibrils, hBMSC were treated with soluble AlexaFluor488-labeled plasma-FN and ATTO655-sHA1 simultaneously. FN was isolated from human plasma (blood transfusion service, Swiss Red Cross, Zurich) using gelatin-sepharose chromatography. Bound FN was eluted from the column with 6 M urea solution, collected and frozen for further studies. For single labeling, a 60-fold molar excess of AlexaFluor488 carboxylic acid succinimidyl ester was used and incubated for 1 h at room temperature (185 μg dye for 2.1 mg FN). Free dye was then excluded by passing the protein solution through a PD-10 column and buffer was changed to PBS.

For monitoring nascent FN fibrils, hBMSC were seeded on 8-well chamber slides in a density of 14,000 cells/cm^2^ to get a confluent monolayer within 24 h after seeding. From 24 h after seeding, hBMSC were treated with 5 μg AlexaFluor488-labeled FN/mL, 45 μg plasma-FN/mL and 200 μg ATTO565-sHA1/mL simultaneously. After 48 h cells were fixed, stained with DAPI and embedded in Mowiol 4–88 as described before.

### Analysis of colocalization

Colocalization of immunofluorescence signals was analyzed with Fiji software using the colocalization plugin JACoP^39^. Threshold was set automatically using Otsu’s method which fitted best for detected signals. Colocalization values (Pearson, Manders’ coefficients)^59^ were calculated from 15 representative images from each of three independent experiments.

### FRET analysis of FN

FN was isolated from human plasma as described before^22^. Cryptic cysteine residues were opened for labeling by denaturation in 4 M guanidine hydrochloride (GdnHCl). For acceptor labeling, a 30-fold molar excess of AlexaFluor546 C5 maleimide (acceptor A) was used and incubated for 1 h at room temperature. Free dye was then excluded by passing the protein solution through a PD-10 column and buffer was exchanged to PBS with 0.1 M NaHCO_3_ at pH 8.5. For donor labeling, a 70-fold molar excess of AlexaFluor488 carboxylic acid succinimidyl ester (donor D) was used to couple free amines and to achieve double labeled FN-DA^21^,^22^. After 1 h of incubation the free dye was again removed by passing the protein solution through a PD-10 column. An average of 7.5 donor labels and 3.5 acceptor labels per FN molecule were calculated for the batch of FN-DA used in this study.

For sample preparation, hBMSC were seeded on 8-well chamber slides in a density of 14,000 cells/cm^2^ to get a monolayer within 24 h. From 2 h after seeding, cells were treated with 5 μg FN-DA/mL and 45 μg plasma-FN/mL. 200 μg GAG/mL (Hep, HA or sHA1) were added 24 h later. After 48 h of incubation with GAG, samples were fixed with 4% (w/v) paraformaldehyde and embedded in Mowiol 4–88.

FRET analysis was performed according to Smith *et al.*^22^. Images were acquired using an Olympus FV-1000 scanning laser confocal microscope equipped with a 40x water objective (NA 0.90). Image analysis was performed using MATLAB based on an adapted version of a previous script^22^. For each sample, 10 randomly chosen fields of view were analyzed. Representative histograms were computed from all data pixels per group. FRET ratios were color-coded to produce FRET images. All FRET ratios (I_A_/I_D_, intensity acceptor/intensity donor) were calibrated to different FN conformations in GdnHCl solution^22^ (0 M GdnHCl (FRET ratio 0.64), 1 M (0.44), 2 M (0.30), 4 M (0.22)).

### FN solubility assay

Cells were seeded in 6 cm petri dishes and treated permanently with 200 μg GAG/mL. At day 8, FN matrix assembly was analyzed using deoxycholate (DOC) solubility as a criterion to distinguish between less and highly assembled FN fibrils^36^. Cells were lysed in 150 μl DOC lysis buffer. DOC-insoluble material was isolated by centrifugation and solubilized afterwards in 40 μl sodium dodecylsulfate (SDS) buffer.

Protein concentration of both fractions was determined by Bradford assay. Equal amounts of total protein of DOC-soluble and DOC-insoluble fractions were reduced with β-mercaptoethanol, boiled for 5 min and resolved by SDS polyacrylamide gel electrophoresis (PAGE). Wet transfer was performed on nitrocellulose membrane and Western blot analysis was performed as previously described^13^ using primary monoclonal mouse anti-human antibody for FN (50 ng/mL) and vimentin (29 μg/mL; Dako Agilent Technologies). Chemiluminescence detection was performed and samples were analyzed densiometrically.

### Analysis of gene expression

RNA was directly prepared from lysates and real time PCR analysis was performed as previously described^11^. Primers were synthesized by Eurofins MWG Operon for *fn1* (114 bp, 5′-3′ sequences: forward CTGGCCGAAAATACATTGTAAA, reversed CCACAGTCGGGTCAGGAG) and ribosomal protein S26 (*rps26*; 189 bp, 5′-3′ sequences: forward CAATGGTCGTGCCAAAAAG, reversed TTCACATACAGCTTGGGAAGC).

### Determination of TNAP activity

TNAP activity was determined from cell lysates as previously described^11^,^12^,^13^. Protein concentration of the lysate was determined separately by Bradford assay and TNAP activity was calculated in mU/mg protein.

### Statistical Analysis

Experiments were performed with non-pooled hBMSC preparations of three or four different bone marrow donors each in duplicate. The results are presented as mean ± standard error of the mean (SEM). Statistical significances were analyzed with one way analysis of variance (ANOVA) with subsequent pairwise post hoc Bonferroni’s test. P values < 0.05 were considered as statistically significant.

## Additional Information

**How to cite this article**: Vogel, S. *et al.* Sulfated hyaluronan alters fibronectin matrix assembly and promotes osteogenic differentiation of human bone marrow stromal cells. *Sci. Rep.* **6**, 36418; doi: 10.1038/srep36418 (2016).

**Publisher’s note**: Springer Nature remains neutral with regard to jurisdictional claims in published maps and institutional affiliations.

## Supplementary Material

Supplementary Information

## Figures and Tables

**Table 1:** Characteristics of synthetic HA derivatives.

HA derivative	HA	ATTO565- HA	sHA1	ATTO565-sHA1	ATTO565- ATTO655-sHA1
D.S.	—	—	1.5	1.0	1.4
fluorescent label [μg/mg]	—	0.47	—	1.40	ATTO655: 0.33 ATTO565: 2.89
M_w_ [g/mol]	84,000	27,000	42,000	27,000	27,000
PD	2.5	2.4	2.3	2.2	1.9

Degree of sulfation, number of sulfate residues per disaccharide unit (D.S.); weight-average (Mw) molecular weight values as determined by gel permeation chromatography equipped with Laser Light Scattering (LLS) detection; molecular weight distributions (polydispersity index: PD) based on the values calculated from RI detection.

## References

[b1] Liang Y. & Kiick K. L. Heparin-functionalized polymeric biomaterials in tissue engineering and drug delivery applications. Acta Biomater. 10, 1588–1600, doi: 10.1016/j.actbio.2013.07.031 (2014). 23911941 PMC3937301

[b2] Pervin A., Gallo C., Jandik K. A., Han X. J. & Linhardt R. J. Preparation and structural characterization of large heparin-derived oligosaccharides. Glycobiology 5, 83–95, doi: 10.1093/glycob/5.1.83 (1995). 7772871

[b3] Verli H. Insights into carbohydrate structure and biological function. Transworld Research Network, 37/661 (2), Nasciemento, F.D. *et al.* Ch. 9 (2006).

[b4] Gallagher J. T., Lyon M. & Steward W. P. Structure and function of heparan sulphate proteoglycans. Biochem. J. 236, 313–325 (1986). 2944511 10.1042/bj2360313 PMC1146843

[b5] Xu D. & Esko J. D. Demystifying heparan sulfate-protein interactions. Annu. Rev. Biochem. 83, 129–157, doi: 10.1146/annurev-biochem-060713-035314 (2014). 24606135 PMC7851832

[b6] Dombrowski C., Song S. J., Chuan P. *et al.* Heparan sulfate mediates the proliferation and differentiation of rat mesenchymal stem cells. Stem Cells Dev. 18, 661–670, doi: 10.1089/scd.2008.0157 (2009). 18690792

[b7] Jackson R. A., Murali S., van Wijnen A. J., Stein G. S., Nurcombe V. & Cool S. M. Heparan sulfate regulates the anabolic activity of MC3T3-E1 preosteoblast cells by induction of Runx2. J. Cell. Physiol. 210, 38–50, doi: 10.1002/jcp.20813 (2007). 17051597

[b8] Nikitovic D., Zafiropoulos A., Tzanakakis G. N., Karamanos N. K. & Tsatsakis A. M. Effects of glycosaminoglycans on cell proliferation of normal osteoblasts and human osteosarcoma cells depend on their type and fine chemical compositions. Anticancer Res. 25, 2851–2856, PMID: 16080537 (2005). 16080537

[b9] Nurcombe V. & Cool S. M. Heparan sulfate control of proliferation and differentiation in the stem cell niche. Crit. Rev. Eukaryot. Gene Expr. 17, 159–171, PMID: 17725486 (2007). 17725486 10.1615/critreveukargeneexpr.v17.i2.50

[b10] Salbach J., Kliemt S., Rauner M. *et al.* The effect of the degree of sulfation of glycosaminoglycans on osteoclast function and signaling pathways. Biomaterials 33, 8418–8429, doi: 10.1016/j.biomaterials.2012.08.028 (2012). 22954516

[b11] Hempel U., Moeller S., Noack C., Hintze V., Scharnweber D., Schnabelrauch M. & Dieter P. Sulfated hyaluronan/collagen I matrices enhance the osteogenic differentiation of human mesenchymal stromal cells *in vitro* even in the absence of dexamethasone. Acta Biomater. 8, 4064–4072, doi: 10.1016/j.actbio.2012.06.039 (2012). 22771456

[b12] Hempel U., Hintze V., Moeller S., Schnabelrauch M., Scharnweber D. & Dieter P. Artificial extracellular matrices with oversulfated glycosaminoglycan derivatives promote the differentiation of osteoblast-precursor cells and premature osteoblasts. BioMed Res. Int. 1–10, doi: 10.1155/2014/938368 (2014). PMC4020545 24864267

[b13] Buettner M., Moeller S., Keller M., Huster D., Schiller J., Schnabelrauch M., Dieter P. & Hempel U. Over-sulfated chondroitin sulfate derivatives induce osteogenic differentiation of hMSC independent of BMP-2 and TGF-β1 signalling. J. Cell. Physiol. 228, 330–340, doi: 10.1002/jcp.24135 (2013). 22718137

[b14] Kliemt S., Lange C., Otto W., Hintze V., Moeller S., von Bergen M., Hempel U. & Kalkhof S. Sulfated hyaluronan containing collagen matrices enhance cell-matrix-interaction, endocytosis, and osteogenic differentiation of human mesenchymal stromal cells. J. Proteome Res. 12, 378–389, doi: 10.1021/pr300640h (2013). 23170904

[b15] Schmidt J. R., Kliemt S., Preissler C., Moeller S., von Bergen M., Hempel U. & Kalkhof S. Osteoblast-released matrix vesicles, regulation of activity and composition by sulfated and non-sulfated glycosaminoglycans. Mol. Cell. Proteomics MCP 15, 558–572, doi: 10.1074/mcp.M115.049718 (2016). 26598647 PMC4739673

[b16] Schofield K. P., Gallagher J. T. & David G. Expression of proteoglycan core proteins in human bone marrow stroma. Biochem. J. 343 Pt 3, 663–668, PMID: 10527946 (1999). 10527946 PMC1220599

[b17] Chen X. D. Extracellular matrix provides an optimal niche for the maintenance and propagation of mesenchymal stem cells. Birth Defects Res. Part C Embryo Today Rev. 90, 45–54, doi: 10.1002/bdrc.20171 (2010). 20301219

[b18] Hynes R. O. Fibronectins, Springer: New York, 1990.

[b19] Bradshaw M. J., Cheung M. C., Ehrlich D. J. & Smith M. L. Using molecular mechanics to predict bulk material properties of fibronectin fibers. PLoS Comput. Biol. 8, e1002845, doi: 10.1371/journal.pcbi.1002845 (2012). 23300425 PMC3531316

[b20] Mao Y. & Schwarzbauer J. E. Fibronectin fibrillogenesis, a cell-mediated matrix assembly process. Matrix Biol. J. Int. Soc. Matrix Biol. 24, 389–399, doi: 10.1016/j.matbio.2005.06.008 (2005). 16061370

[b21] Baneyx G., Baugh L. & Vogel V. Coexisting conformations of fibronectin in cell culture imaged using fluorescence resonance energy transfer. Proc. Natl. Acad. Sci. USA. 98, 14464–14468, doi: 10.1073/pnas.251422998 (2001). 11717404 PMC64704

[b22] Smith M. L., Gourdon D., Little W. C., Kubow K. E., Eguiluz R. A., Luna-Morris S. & Vogel V. Force-induced unfolding of fibronectin in the extracellular matrix of living cells. PLoS Biol. 5, e268, doi: 10.1371/journal.pbio.0050268 (2007). 17914904 PMC1994993

[b23] Vogel V. Mechanotransduction involving modular proteins: converting force into biochemical signals. Annu. Rev. Biophys. Biomol. Struct. 35, 459–488, doi: 10.1146/annurev.biophys.35.040405.102013 (2006). 16689645

[b24] Frueh S. M., Schoen I., Ries J. & Vogel V. Molecular architecture of native fibronectin fibrils. Nat. Commun. 6, 7275, doi: 10.1038/ncomms8275 (2015). 26041410 PMC4468872

[b25] Schwarzbauer J. E. & DeSimone D. W. Fibronectins, their fibrillogenesis, and *in vivo* functions. Cold Spring Harb. Perspect. Biol. 3, a005041, doi: 10.1101/cshperspect.a005041 (2011). 21576254 PMC3119908

[b26] Singh P., Carraher C. & Schwarzbauer J. E. Assembly of fibronectin extracellular matrix. Annu. Rev. Cell Dev. Biol. 26, 397–419, doi: 10.1146/annurev-cellbio-100109-104020 (2010). 20690820 PMC3628685

[b27] Kadler K. E., Hill A. & Canty-Laird E. G. Collagen fibrillogenesis: fibronectin, integrins, and minor collagens as organizers and nucleators. Curr. Opin. Cell Biol. 20, 495–501, doi: 10.1016/j.ceb.2008.06.008 (2008). 18640274 PMC2577133

[b28] Sottile J. Fibronectin polymerization regulates the composition and stability of extracellular matrix fibrils and cell-matrix adhesions. Mol. Biol. Cell 13, 3546–3559, doi: 10.1091/mbc.E02-01-0048 (2002). 12388756 PMC129965

[b29] Sekiguchi K., Hakomori S. I., Matsumoto S. I., Seno I. & Binding N. of fibronectin and its proteolytic fragments of glycosaminoglycans: Exposure of cryptic glycosaminoglycan-binding domains upon limited proteolysis. J. Biol. Chem. 258, 143859–14365, PMID: 6643486 (1983a). 6643486

[b30] Yamada K. M., Kennedy D. W., Kimata K. & Pratt R. M. Characterization of fibronectin interactions with glycosaminoglycans and identification of active proteolytic fragments. J. Biol. Chem. 255, 6055–6063, PMID: 6771264 (1980). 6771264

[b31] Mitsi M., Hong Z., Costello C. E. & Nugent M. A. Heparin-mediated conformational changes in fibronectin expose vascular endothelial growth factor binding sites. Biochemistry (Mosc.) 45, 10319–10328, doi: 10.1021/bi060974p (2006). 16922507

[b32] Chatakun P., Nunez-Toldra R., Diaz Lopez J. *et al.* The effect of five proteins on stem cells used for osteoblast differentiation and proliferation: a current review of the literature. Cell. Mol. Life Sci. 71, 113–142, doi: 10.1007/s00018-013-1326-0 (2014). 23568025 PMC11113514

[b33] Di Benedetto A., Brunetti G., Posa F. *et al.* Osteogenic differentiation of mesenchymal stem cells from dental bud: Role of integrins and cadherins. Stem Cell Res. 15, 618–628, doi: 10.1016/j.scr.2015.09.011 (2015). 26513557

[b34] Mathews S., Bhonde R., Gupta P. K. & Totey S. Extracellular matrix protein mediated regulation of the osteoblast differentiation of bone marrow derived human mesenchymal stem cells. Differentiation 84, 185–192, doi: 10.1016/j.diff.2012.05.001 (2012). 22664173

[b35] Li B., Lin Z., Mitsi M., Zhang Y. & Vogel V. Heparin-induced conformational changes of fibronectin within the extracellular matrix promote hMSC osteogenic differentiation. Biomater Sci 3, 73–84, doi: 10.1039/C3BM60326A (2015). 26214191

[b36] Wierzbicka-Patynowski I., Mao Y. & Schwarzbauer J. E. Analysis of fibronectin matrix assembly. J Cell Science 116, 3269–3276, doi: 10.1242/jcs.00670 (2003). 12857786

[b37] Christianson H. C., Svensson K. J. & Belting M. Exosome and microvesicle mediated phene transfer in mammalian cells. Semin. Cancer Biol. 28, 31–38, doi: 10.1016/j.semcancer.2014.04.007 (2014). 24769057

[b38] Lu H., Hoshiba T., Kawazoe N. & Chen G. Comparison of decellularization techniques for preparation of extracellular matrix scaffolds derived from three-dimensional cell culture. J. Biomed. Mater. Res. 100A(9), 2507–2516, doi: 10.1002/jbm.a.34150 (2012) 22623317

[b39] Bolte S. & Cordelières F. P. A guided tour into subcellular colocalization analysis in light microscopy. J. Microsc. 224, 213–232, doi: 10.1111/j.1365-2818.2006.01706.x (2006). 17210054

[b40] Sharma A., Askari J. A., Humphries M. J., Jones E. Y. & Stuart D. I. Crystal structure of a heparin- and integrin-binding segment of human fibronectin. EMBO J. 18, 1468–1479, doi: 10.1093/emboj/18.6.1468 (1999). 10075919 PMC1171236

[b41] Hubbard B., Buczek-Thomas J. A., Nugent M. A. & Smith M. L. Heparin-dependent regulation of fibronectin matrix conformation. Matrix Biol. 34, 124–131, doi: 10.1016/j.matbio.2013.10.006 (2014). 24148804 PMC3992196

[b42] Johnson K. J., Sage H., Briscoe G. & Erickson H. P. The compact conformation of fibronectin is determined by intramolecular ionic interactions. J. Biol. Chem. 274, 15473–15479, PMID: 10336438 (1999). 10336438 10.1074/jbc.274.22.15473

[b43] Pittenger M. F. Mesenchymal stem cells from adult bone marrow, Methods and protocols mesenchymal stem cells, Methods in molecular biology, Humana Press Springer, Prockop D. J., Bunnell B. A., Phinney D. G. Vol 449, 27–44 (2008). 10.1007/978-1-60327-169-1_2 18370081

[b44] Mitsi M., Forsten-Williams K., Gopalakrishnan M. & Nugent M. A. A Catalytic Role of Heparin within the Extracellular Matrix. J. Biol. Chem. 283, 34796–34807, doi: 10.1074/jbc.M806692200 (2008). 18845539 PMC2596404

[b45] Becher J., Moeller S. & Schnabelrauch M. Phase transfer-catalyzed synthesis of highly acrylated hyaluronan. Carbohydr. Polym. 93, 438–441, doi: 10.1016/j.carbpol.2012.12.056 (2013). 23499080

[b46] Esko J. D. & Lindahl U. Molecular diversity of heparan sulfate. J. Clin. Invest. 108, 169–173, doi: 10.1172/JCI13530 (2001). 11457867 PMC203033

[b47] Chen H. & Mosher D. F. Formation of sodium dodecyl sulfate-stable fibronectin multimers. Failure to detect products of thiol-disulfide exchange in cyanogen bromide or limited acid digests of stabilized matrix fibronectin. J. Biol. Chem. 271, 9084–9089 (1996). 8621558 10.1074/jbc.271.15.9084

[b48] Cui C., Wang S., Myneni V. D., Hitomi K. & Kaartinen M. T. Transglutaminase activity arising from Factor XIIIA is required for stabilization and conversion of plasma fibronectin into matrix in osteoblast cultures. Bone 59, 127–138, doi: 10.1016/j.bone.2013.11.006 (2014). 24246248

[b49] Langenbach K. J. & Sottile J. Identification of protein-disulfide isomerase activity in fibronectin. J. Biol. Chem. 274, 7032–7038, PMID: 10066758 (1999). 10066758 10.1074/jbc.274.11.7032

[b50] Peysselon F., Xue B., Uversky V. N. & Ricard-Blum S. Intrinsic disorder of the extracellular matrix. Mol. Biosyst. 7, 3353, doi: 10.1039/c1mb05316g (2011). 22009114

[b51] Klotzsch E., Smith M. L., Kubow K. E. *et al.* Fibronectin forms the most extensible biological fibers displaying switchable force-exposed cryptic binding sites. Proc. Natl. Acad. Sci. 106, 18267–18272, doi: 10.1073/pnas.0907518106 (2009). 19826086 PMC2761242

[b52] Sechler J. L., Rao H., Cumiskey A. M., Vega-Colón I., Smith M. S., Murata T. & Schwarzbauer J. E. A novel fibronectin binding site required for fibronectin fibril growth during matrix assembly. J. Cell Biol. 154, 1081–1088, doi: 10.1083/jcb.200102034 (2001). 11535624 PMC2196193

[b53] Ling L., Camilleri E. T., Helledie T. *et al.* Effect of heparin on the biological properties and molecular signature of human mesenchymal stem cells. Gene 576, 292–303, doi: 10.1016/j.gene.2015.10.039 (2016). 26484394 PMC5330685

[b54] Hintze V., Moeller S., Schnabelrauch M., Bierbaum S., Viola M., Worch H. & Scharnweber D. Modifications of Hyaluronan Influence the Interaction with Human Bone Morphogenetic Protein-4 (hBMP-4). Biomacromolecules 10, 3290–3297, doi: 10.1021/bm9008827 (2009). 19894734

[b55] Kunze R., Roesler M., Moeller S., Schnabelrauch M., Riemer T., Hempel U. & Dieter P. Sulfated hyaluronan derivatives reduce the proliferation rate of primary rat calvarial osteoblasts. Glycoconj. J. 27, 151–158, doi: 10.1007/s10719-009-9270-9 (2010). 19941065

[b56] Rother S., Salbach-Hirsch J., Moeller S., Seemann T., Schnabelrauch M., Hofbauer L. C., Hintze V. & Scharnweber D. Bioinspired Collagen/Glycosaminoglycan-Based Cellular Microenvironments for Tuning Osteoclastogenesis. ACS Appl. Mater. Interfaces 7, 23787–23797, doi: 10.1021/acsami.5b08419 (2015). 26452150

[b57] Salbach-Hirsch J., Ziegler N., Thiele S., Moeller S., Schnabelrauch M., Hintze V., Scharnweber D., Rauner M. & Hofbauer L. C. Sulfated Glycosaminoglycans Support Osteoblast Functions and Concurrently Suppress Osteoclasts: Osteogenic Properties of GAG Sulfation. J. Cell. Biochem. 115, 1101–1111, doi: 10.1002/jcb.24750 (2014). 24356935

[b58] Oswald J., Boxberger S., JØrgensen B., Feldmann S., Ehninger G., Bornhaeuser M. & Werner C. Mesenchymal stem cells can be differentiated into endothelial cells *in vitro*. Stem Cells Dayt. Ohio 22, 377–384, doi: 10.1634/stemcells.22-3-377 (2004). 15153614

[b59] Zinchuk V., Zinchuk O. & Okada T. Quantitative colocalization analysis of multicolor confocal immunofluorescence microscopy images: pushing pixels to explore biological phenomena. Acta Histochem. Cytochem. 40, 101–111, doi: 10.1267/ahc.07002 (2007). 17898874 PMC1993886

