# Supplementary material for: Sulfated hyaluronan alters fibronectin matrix assembly and promotes osteogenic differentiation of human bone marrow stromal cells
Source: Sci Rep. 2016 Nov 3;6:36418. doi: 10.1038/srep36418 (PMC5093463; doi:10.1038/srep36418)
Supplement: Supplementary Information [file srep36418-s1.pdf]

Supplementary information

Sulfated hyaluronan alters fibronectin matrix assembly and promotes osteogenic differentiation of human bone marrow stromal cells

Sarah Vogel[1], Simon Arnoldini[2], Stephanie Möller[3], Matthias Schnabelrauch[3], Ute Hempel[1*]

[1]Institute of Physiological Chemistry, Carl Gustav Carus Faculty of Medicine, Technische Universität Dresden, Fiedlerstrasse 42, 01307 Dresden, Germany, sarah.vogel@tu-dresden.de, ute.hempel@tu-dresden.de

[2]Laboratory of Applied Mechanobiology, Department of Health Science and Technology, ETH Zurich, Vladimir-Prelog-Weg 4, 8093 Zurich, Switzerland, simon.arnoldini@hest.ethz.ch

[3]Biomaterials Department, INNOVENT e. V., Pruessingstrasse 27B, 07745 Jena, Germany, SM@innovent-jena.de, MS@innovent-jena.de

*Corresponding author: ute.hempel@tu-dresden.de

This file includes further explanantion and supplementary figures:

Fig. S1

Fig. S2

Fig. S3.

A quantitative colocalization analysis of the two dyes of ATTO565-ATTO655-sHA1 derivative confirmed nearby complete colocalization of ATTO565 and ATTO655 (Fig. S1).

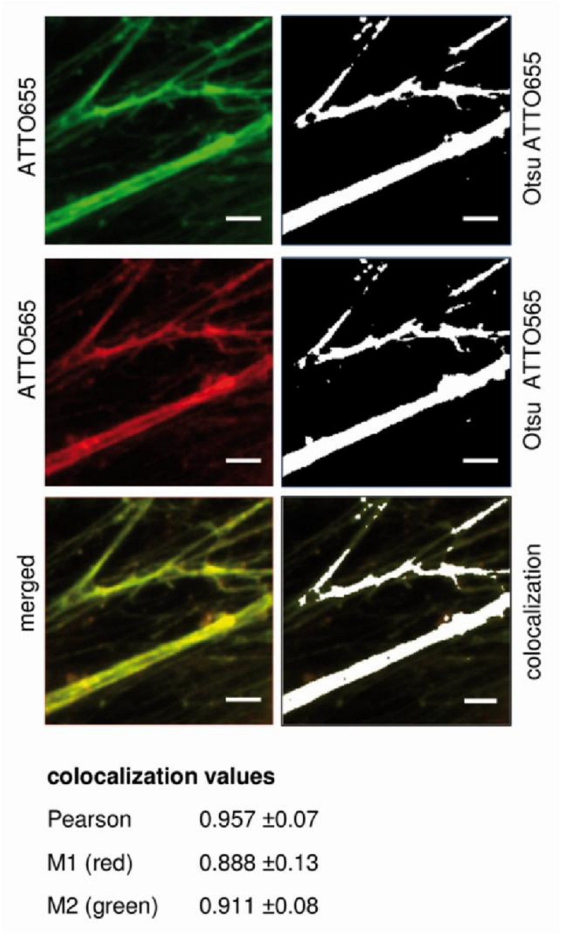

Fig. S1: Colocalization analysis of double-labeled ATTO565-ATTO655-sHA1. hBMSC were cultured on TCPS, treated with 200 µg ATTO565-ATTO655-sHA1/mL from day 1 until day 8. At day 8 samples were washed and fixed. Images were taken using the corresponding filter set as given in materials and methods section. For better discrimination the ATTO655 (far red channel) is presented in green (left panel). Scale bar 5 µm. Yellow fluorescence indicated that both fluorescence labels stayed in close vicinity suggesting intact sHA1 molecules. Images were visualized in false colors after approaching Otsu filter (right panel; signal in white, background in black) and analyzed in JACoP Plugin in Fiji Software. The summary of colocalization values of 15 randomly chosen images (values as mean ±SEM) is shown underneath.

The relative FN protein content of cell lysates was analyzed by Western blotting (representative cropped blots in Fig. 5A and analysis in Fig. 5B-C). Representative full length Western blots (total membrane width) are shown in Fig. S2.

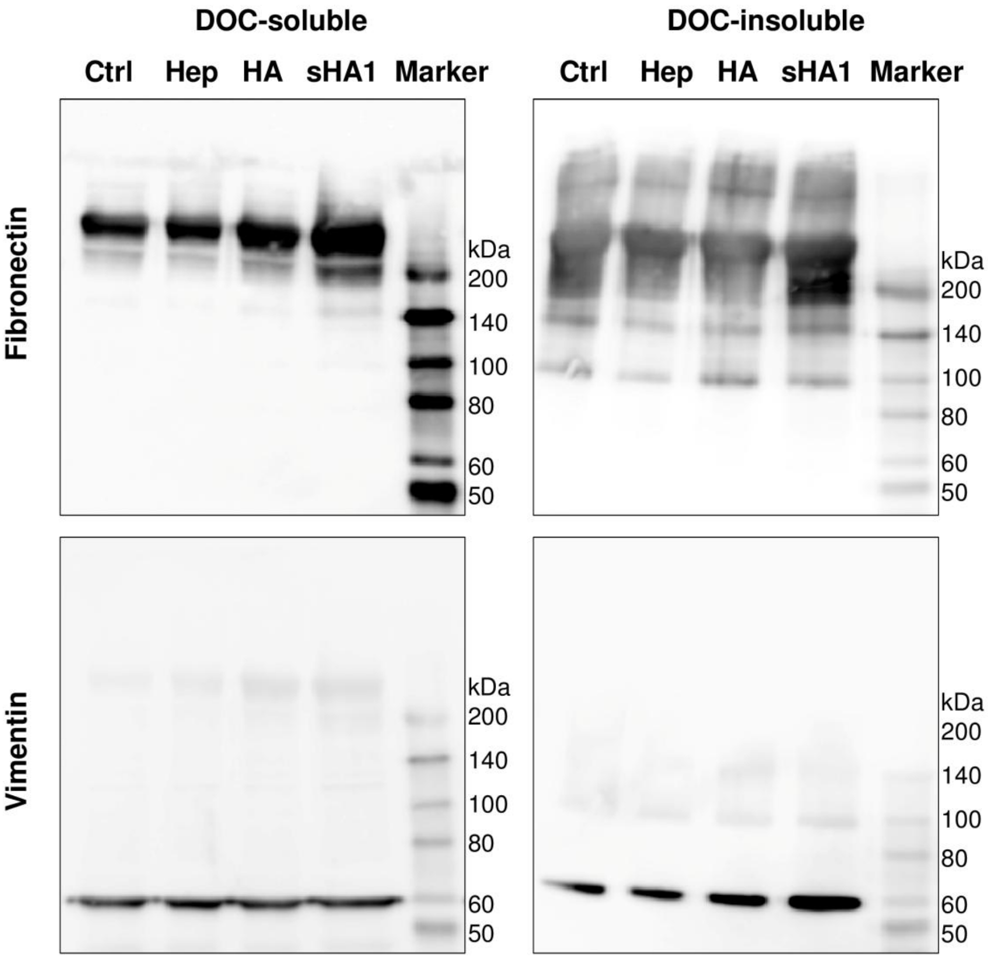

Fig. S2: Representative full length Western blots of DOC-soluble and DOC-insoluble fractions. Cell lysates derived from the same experiment and both gels/blots were processed in parallel.

The impact of GAG derivatives on TNAP activity of hBMSC was also demonstrated in osteogenic medium (Fig. S3) and showed similar results like in basal medium without osteogenic supplements (see Fig. 6).

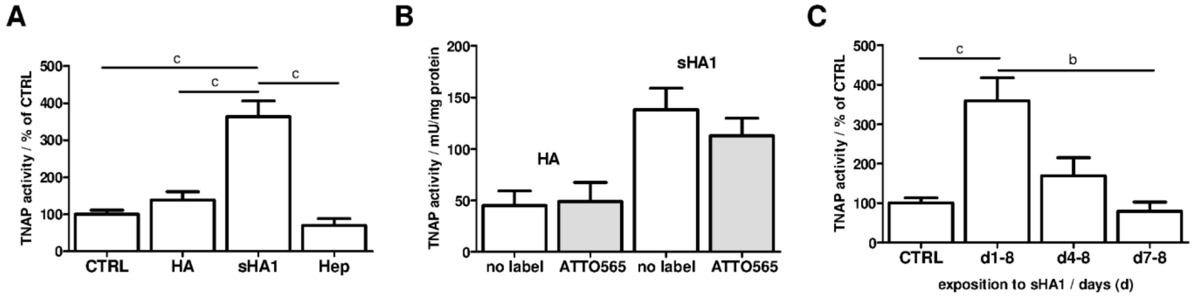

Fig. S3: Impact of GAG derivatives on TNAP activity in osteogenic medium. hBMSC were cultured on TCPS (CTRL) and treated with dissolved GAG derivatives as described. Medium exchange was performed at day 4 and osteogenic supplements were added. TNAP activity as determined from cell lysates at day 8. Diagrams show calculated values as mean ±SEM. **A**) hBMSC were treated with 200 µg GAG derivative/mL for 8 days. Diagram shows TNAP activities calculated from five different donors. Untreated control (CTRL) was set to 100%. Significant differences between sHA1 and CTRL, HA or Hep are indicated with c ($p<0.001$) or b ($p< 0.01$). **B**) hBMSC were treated with 200 µg HA or sHA1/mL or with the equivalent ATTO565-labeled derivative for 8 days. n=3. **C**) hBMSC were exposed to 1, 10, 50 or 200 µg sHA1/mL for 8 days. TNAP activities were calculated from three different donors and untreated control (CTRL, 0 µg sHA1/mL) is indicated with dotted line. **D**) hBMSC were exposed to 200 µg sHA1/mL for different time frames (d1-8, d4-8, d7-8). TNAP activities were calculated from three different donors and untreated control (CTRL) was set to 100%. Significant differences between (d1-8) and CTRL, (d4-8) or (d7-8) are indicated with c ($p<0.001$) or a ($p<0.05$).
